# Challenges to women’s cancer control in Morocco: a qualitative study of lay advisors and civil society perspectives

**DOI:** 10.1017/S1463423625100169

**Published:** 2025-06-16

**Authors:** Amy Luo, Maha Naamaoui, Amr Soliman, Majdouline Obtel, Wafaa Kaikani, Hafida Charaka, Chakib Nejjari, Mohamed Khalis

**Affiliations:** 1 Department of Global Health and Population, Harvard School of Public Health, Boston, MA, USA; 2 Department of Population, Family and Reproductive Health, Johns Hopkins School of Public Health, Baltimore, MD, USA; 3 Faculty of Medicine, Mohammed VI University of Health Sciences (UM6SS), Casablanca, Morocco; 4 Community Health and Social Medicine Department, CUNY School of Medicine, The City College of New York, New York, NY, USA; 5 Laboratory of Biostatistics, Clinical, and Epidemiological Research, Faculty of Medicine and Pharmacy, Department of Public Health, Mohamed V University in Rabat, Rabat, Morocco; 6 Department of Oncology, Faculty of Medicine, Mohammed VI University of Health Sciences (UM6SS), Casablanca, Morocco; 7 Higher Institute of Nursing Professions and Health Techniques, Ministry of Health and Social Protection, Rabat, Morocco; 8 Euromed Research Center, Euromed University of Fez, Fez, Morocco; 9 Faculty of Medicine, Pharmacy and Dental Medicine, University Sidi Mohammed Ben Abdellah, Fez, Morocco; 10 Department of Public Health and Clinical Research, Mohammed VI Center for Research and Innovation, Rabat, Morocco; 11 Mohammed VI International School of Public Health, Mohammed VI University of Sciences and Health (UM6SS), Casablanca, Morocco

**Keywords:** cancer, community health, health systems, lay health providers, Morocco

## Abstract

**Aim:**

This study explores the perspectives of cancer lay health providers and civil society on the barriers and facilitators to cancer detection and treatment among women.

**Background:**

In 2010, the Moroccan Ministry of Health implemented a national plan for cancer care and control. Activities focused on strengthening multisectoral collaboration in cancer care and control, including promoting early detection in primary care. Despite progress in reducing women’s cancer mortality, socio-cultural challenges impede further gains. Elucidating the perspectives of the community-based and civil society allied in cancer control is critical to addressing cancer disparities.

**Methods:**

Data were collected through in-depth interviews with cancer lay health advisors (*n* = 10) and civil society members (*n* = 10) on topics of challenges and opportunities to improve care-seeking and treatment. Data were analysed using thematic analysis and guided by the socio-ecological model.

**Findings:**

Barriers and facilitators to early diagnosis and treatment were identified at levels of the individual, family, community/societal, and the health system. Barriers to early detection include taboo and stigma, fear of death, and gender norms and roles. Financial and geographic barriers, lack of psychosocial support, and poor health system/provider communication were major deterrents related to treatment. Results suggest intervention targets to reduce late-stage presentation for women, including enhancing educational efforts and augmenting community outreach linkages to primary care.

## Introduction

Cancer ranks as a leading cause of disability and deaths among women globally. Approximately one in five women will develop cancer during their lifetime, and one in 12 women die from it (Bray *et al.*, 2024). Cancer is associated with profound public health, societal, and economic impacts, including both the direct costs of treatment and indirect costs associated with the loss of productivity and disruptions to families and communities due to caregiving, illness, and premature death (Chen *et al.*, 2023; Guida *et al.*, 2022). Despite cancer being a universal public health concern, there are striking disparities in women’s cancer burden across regions and countries worldwide. The burden of cancer is significantly higher in low- and middle-income countries (LMICs). Both demographic (population ageing) and epidemiological transitions (increasing burden of non-communicable diseases related to lifestyle behavioural changes) in combination with inadequate healthcare resources contribute to disproportionate cancer morbidity and mortality in LMICs (Ginsburg *et al.*, 2017). For instance, up to 30% of women with breast cancer were diagnosed at late metastatic stages in LMICs compared to 10% in Europe and North America, and nearly 94% of deaths due to cervical cancer occurred in LMICs (Benitez Fuentes *et al.*, 2024; Ferlay *et al.*, 2024).

Morocco, a lower-middle-income country, is undergoing this demographic and epidemiologic transition, with corresponding increasing cancer rates among women (Belbaraka *et al.*, 2022; Safiri *et al.*, 2022). Cancer ranks as the second leading cause of death among women in Morocco, and the incidence of the top five cancer sites in women has increased by approximately 20% between 2012 and 2022 (GLOBOCAN, 2022; Royaume du Maroc, 2015; Selmouni *et al.*, 2018). To address this issue, the Moroccan Ministry of Health established the 2010–2019 National Cancer Prevention and Control Plan (NCPCP). The NCPCP sets priorities and strategic actions to coordinate cancer control and prevention measures for common cancers, such as the implementation of a nationwide breast screening programme (Mrabti *et al.*, 2021). As mounting evidence suggests, national cancer control plans can synchronize national health priorities, financing, and intervention delivery when developed and implemented effectively (Romero *et al.*, 2018; 2025). Among LMICs, Morocco has emerged as an exemplar (Selmouni *et al.*, 2018; Oar *et al.*, 2019).

To date, Morocco has achieved substantial improvements in increasing the number of trained healthcare workforce and specialized care facilities. Between 2010 and 2019, approximately 3,400 primary healthcare providers were trained in cancer screening (Khalis *et al.*, 2021). The Ministry of Health has made significant investments in improving diagnostic and treatment facilities, such as the number of dedicated cancer early detection and specialized treatment centres. In addition, national education campaigns – including dedication of October as a women’s cancer awareness month – have promoted lifestyle changes, recognition of cancer warning signs, and uptake of regular cancer screening (Mrabti *et al.*, 2021). Despite these strategic efforts, cancer registry data suggest mixed improvements in early detection and treatment, citing barriers such as socioeconomic, cultural, geographic factors as well as barriers such as access to care and low awareness (Arechkik *et al.*, 2022; Piñeros *et al.*, 2022; Soliman *et al.*, 2019).

Increasingly, innovative solutions to cancer control and care have underscored the importance of partnerships. Strategies that combat entrenched socio-cultural barriers – such as cancer stigma and shame, while strengthening health networks and promoting women’s leadership have been well-recognized in meeting the rising burden of women’s cancers (Rodriguez *et al.*, 2017; Bachelet, 2017; Ginsburg *et al.*, 2017). One strategy includes expanding cancer lay health advisor (LHA) programmes. LHA are trained volunteers or workers that help promote health and disease prevention among communities that have traditionally lacked access to adequate care (Earp *et al.*, 1997). As they often work in communities they are a part of, LHAs are valued members of the healthcare workforce because of their understanding of community needs, values, and cultures to communicate and connect community members to care (Rhodes *et al.*, 2007). Other stakeholders in NCCP implementation include cancer civil society advocacy groups. Here, partners are recognized for their key role, only second to Ministries of Health as reported in a global analysis of NCCPs (Romero *et al.*, 2025). In Morocco, civil society groups played a pivotal role in establishing cancer as a major public health priority. For instance, the Lalla Salma Foundation for Cancer Prevention and Treatment has met significant milestones in advocating for and mobilizing programmes for cancer prevention, detection, and treatment (Khalis *et al.*, 2021).

Previous literature examining the factors associated with screening, detection, and treatment among Moroccan women remain limited to the perspectives of cancer patients and healthcare providers (Charaka *et al.*, 2019; Ghanem *et al.*, 2011; Maghous *et al.*, 2016; Soliman *et al.*, 2019). These studies highlighted the importance of addressing structural and socio-cultural barriers that contribute to delayed diagnosis and treatment for women’s cancers, emphasizing the need for broader cancer prevention education and advocacy within the community. However, to date, no study has examined the perspectives of LHAs and civil society members, who often serve as primary community-based cancer educators and advocates. This study aimed to identify the barriers and facilitators to early cancer diagnosis and treatment for women in Morocco from the perspectives of LHAs and cancer civil society members – all of whom are cancer survivors.

## Methods

### Study design

This qualitative study employed a descriptive phenomenological design to semi-structured in-depth interview. Fundamental aspects of this design support the explorations of the lived experience, perceptions, and personal meanings of those interviewed.

### Study area

Morocco is located in the Middle East and North Africa with a population of approximately 38 million people in 2024 (United Nations, Department of Economic and Social Affairs, Population Division, 2024). Morocco has relatively younger population, with a median age of 29.8 years (United Nations, Department of Economic and Social Affairs, Population Division, 2024). As aforementioned, population ageing alongside the increasing burden of non-communicable diseases is an increasing public health concern for this lower-middle-income country. This study took place in the city of Casablanca – the largest urban centre in Morocco and the most populous city in North African Maghreb region. Casablanca houses one of Morocco’s two largest public-funded oncology centres, namely the Centre Mohammed VI Pour le Traitement des Cancers. Affiliated with the Ibn Rochd University Hospital, the specialized cancer treatment centre offers comprehensive medical and surgical care for gynaecological and breast cancers among other cancers for residents of Casablanca and neighbouring regions.

### Study population

In 2021, the Mohammed VI University of Health Sciences in Casablanca, Morocco implemented the first cancer LHA training programme, termed the Patient-Partners programme. Enrolment in this free-of-charge programme was open to cancer survivors, regardless of experience, and age. The objective of this programme was to empower cancer survivors to become trained patient and community-based advocates of advancing cancer control and care. From June to August 2021, the training programme combined didactic and experiential learning methods through modules on cancer biology and treatment, patient communication and support, and patient medical and legal rights. Students applied training modules by proposing and conducting independent projects in cancer care and advocacy under the guidance of university faculty. The goal of this inaugural LHA cohort was to provide holistic and formal training in cancer care such that they may serve as volunteer-based patient advocates, navigators, and community supports in cancer screening, detection, treatment, and palliative care.

To complement the perspectives of participants of the Patient-Partner programme, this study included women involved in cancer civil society. In Morocco, cancer civil society and non-governmental organizations play an important role as partners in cancer control efforts. Organizations such as the Lalla Salma Foundation and Les Amis du Ruban Rose fill gaps in cancer care. Services provided encompass a wide range, including psychosocial and financial support for women undergoing cancer treatment, and national education campaigns. Engaging the perspectives of cancer civil society is essential to raise the visibility of the experiences and perspectives of non-health system actors in the women’s cancer space.

### Sampling

LHAs were selected using convenience sampling. All women in the Patient-Partner programme who were actively engaged in cancer advocacy work were invited to participate in this study. As per the requirements of the Patient-Partner programme, all participants were cancer survivors, adding to the rich exploration of multifaceted lived experiences. In addition, women involved in cancer civil society were purposively sampled using a stratified approach to include perspectives from multiple cancer civil society organizations. Likewise, only civil society members who shared experiences as former cancer patients were eligible to participate.

Participant groups were recruited in two ways. To recruit the cancer lay health workers, an introductory session with all eligible women was held to introduce study aims and what participation entails. Those interested were contacted over the phone or social media (WhatsApp) to schedule an interview. Members of cancer civil society were recruited under important safeguards to respect the privacy of individual health history while encouraging diversity of information-rich cases. After consent to be contacted was obtained, the research assistant contacted interested women via phone or social media (WhatsApp) to arrange an interview time. The final sample size was determined based on the point of saturation, or when no new themes or information was acquired from the data.

### Data collection

Data were collected through individual in-depth interviews using a semi-structured interview guide. Previous studies on women’s cancer barriers and facilitators and the cancer care continuum and coauthors’ expertise informed the topic guide (Brand *et al.*, 2019; Soliman *et al.*, 2019). Topic guides were tailored to the participant group and open-ended questions were broad to capture the richness of participant experiences. Questions were organized to explore four main categories: personal experience in cancer work, barriers and facilitators for women, multilevel factors in the cancer care continuum, and participant sociodemographic characteristics (Appendix 1). Barriers are defined as any factor that prevents, hinders, or limits a patient from seeking medical care or continuing with treatment, whereas facilitators include any supporting, favouring, or helpful determinants of positive healthcare behaviours.

Topic guide content and translation to Arabic and French were collaboratively refined with coauthors before pilot cognitive testing. Pilot participants were asked to provide their comprehension of interview questions and any suggestions to improve the content or question structure. Results from the two pilot interviews assessed interview guide’s reliability and validity and were incorporated in the final interview guide.

All interviews were conducted between July and August 2021 by a coauthor trained in qualitative research and fluent in Arabic, French, and English (MN). In-depth interviews ranged from 30 to 45 min and were conducted over the phone given the COVID-19 pandemic precautions in the participants’ preferred language, Arabic or French. Interviews were structured by the topic guide; probing questions allowed for elaboration on or clarification of answers. Notes and observations were recorded immediately following each interview by the interviewer.

Audio recordings of the in-depth interviews were transcribed and translated to English by the research assistant who conducted the interviews. During data collection, the research team collectively debriefed weekly over online conference calls to discuss progress, challenges, and emerging findings. The quality of translated transcripts and interview content was iteratively assessed, allowing to discuss and correct for any discrepancies during data collection.

### Data analysis

**Figure 1.**
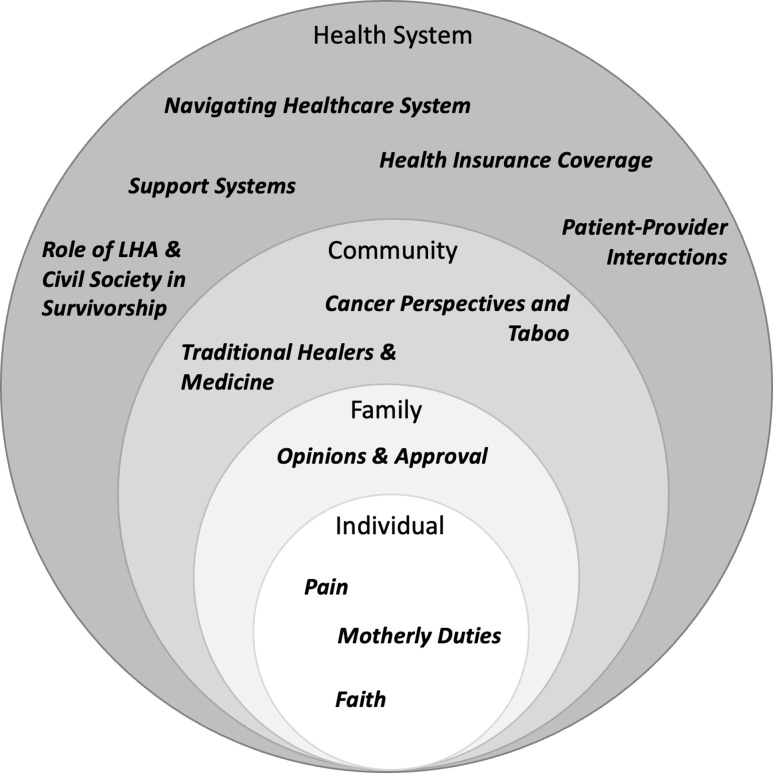
Socio-ecological model of the barriers and facilitators to women’s cancer care in Morocco.

Data were analysed using a thematic analysis approach, selected for its inherent flexibility in identifying patterns within the perspectives shared (Braun and Clarke, 2006). Analysi, informed by the socio-ecological model of health, categorized themes according to individual, familial, community or societal, and health system levels (Bronfenbrenner, 1977) (Figure 1). The initial codebook was developed based on a review of two transcripts – one per each participant group – until consensus was reached by two coauthors (AL & MN) before discussion and refinement with all coauthors. Remaining transcripts and observational notes were divided and independently coded, annotated, and analysed in NVivo Version 12 (AL & MN) The codebook was iteratively refined during analysis and bi-weekly meetings resolved any disagreements. To assess intercoder reliability, one transcript not coded by the research assistant was randomly selected and compared line-by-line. Codes were reviewed to generate themes and through a consensus-building process until thematic saturation. To improve credibility and validity, results were triangulated with observations collected at the time of interviews and an audit trail documenting data, methods, decisions, and progress. Objectivity was enhanced by reviewing the final themes with the research team.

### Ethical considerations

Ethics approval was obtained from the Institutional Review Board of the Harvard T.H. Chan School of Public Health (IRB19-0620) and locally by the Ethics Review Board of the Mohammed VI University of Health Sciences in Casablanca, Morocco. Verbal informed consent, in Arabic or French, was obtained before each interview, with the option of opting out of audio recording. Participants were recruited in July 2021.

### Reflexivity

The study team comprised of a collaborative and multidisciplinary team. Study site team members contributed their extensive expertise with the Moroccan health system, cancer care continuum, and the socio-cultural context. Data collection by a female medical student (MN), with experiential knowledge and prior working experience in medical care at the study site, was essential in building rapport with participants and contextualizing the participant experience during the interview guide design and coding process. The study was advised by Moroccan public health professors and oncologists with extensive expertise researching cancer epidemiology and designing innovative interventions – including close involvement in the implementation of the Patient-Partner programme (MO, WK, HC, CN & MK). United States-based authors (AL & AS) have extensive qualitative experience in global health, cancer, and women’s health. Both authors have previously lived and conducted research in Morocco. Overall, the variety of expertise, experiences, and cultural perspectives, from study conception to completion, facilitated a thorough and balanced interpretation of the data.

## Results

### Sociodemographic characteristics

Table 1 describes the characteristics of Patient-Partner and members of civil society interviewed. The mean age of participants was 45 years, and most participants were married (75%) and had higher than bachelor’s degree education (61.1%). The most common cancer sites included breast (53.8%), leukaemia (23.1%), lymphoma (7.7%), and rectal (7.7%) cancers.

**Table 1.** Demographic characteristics of participants

Characteristic	Number of participants (%)
Age, years (Mean: 45, *n* = 19)	
30–39	5 (26.3)
40–49	7 (36.8)
50–59	7 (36.8)
Marital status	
Married	15 (75.0)
Single	2 (10.0)
Divorced	2 (10.0)
Widowed	1 (5.0)
Education attainment [*n* = 18]	
Less than secondary	1 (5.6)
Secondary	1 (5.6)
High School	1 (5.6)
Bachelors	4 (22.2)
Higher	11 (61.1)
Cancer Site [*n* = 13]	
Breast	7 (53.8)
Leukaemia	3 (23.1)
Lymphoma	1 (7.7)
Rectal	1 (7.7)

### Barriers and facilitators to cancer care-seeking and treatment

The following sections describe the barriers and facilitators to cancer care-seeking and treatment. Results are organized into four levels, as described by the socio-ecological model: individual, familial, community/societal, and health system (healthcare cascade, insurance, social security, and government partners) (Figure 1).

### Individual level

#### Pain as the main driver to seeking care

When asked how help-seeking decisions were made, participants provided complex and multifaceted answers about when they personally felt to be acceptable to seek care. Often, this decision required their concern for their health to outweigh other non-health priorities. Internal conflicts led to negative coping, including the normalization of symptoms, poor recognition of cancer symptoms, and the anticipated spousal disapproval or rejection. Severe physical symptoms, such as unmanageable pain, heavy bleeding, and nodule palpitation, were commonly cited as turning points that prompted women to seek medical. Despite the severity of symptoms, participants mentioned that symptoms alone were often insufficient to overcome the hesitation and fear associated with seeking medical care.

‘We don’t go to the doctor directly in our region unless we are really hurting… If they palpate a node for instance in their breast, they go to the doctor, and before that, never. And even if they do, they sometimes do it secretly without telling the husband’ (*Participant 201*)

Particularly in rural regions, late presentation to care was exacerbated by a lack of awareness, education, and access to nearby health services. Even among participants who were aware of cancer alarm symptoms, knowledge of non-specific cancer symptoms (e.g., weight loss, fatigue, pale complexion) was generally poor. Before seeking care from a healthcare provider, participants first disclosed their symptoms to family members, relatives, and friends – especially other women – often facilitated via social media (WhatsApp).

#### Motherly duties

Self-resilience and perseverance during treatment were strongly influenced by a woman’s role as a mother. Participants reflected on how significant their love for their children and motherly duties gave them strength to seek and continue treatment. However, this same role also hindered their decisions to seek medical care upon symptom presentation. While the fear of leaving their children and their deep maternal love motivated them to recover and persevere after a cancer diagnosis, it also intensified their fear of their illness and feelings of uncertainty.

‘When I was sick, I dreamed of death every night, I dreamed of my coffin. I was afraid for my children, who will replace me? Will she know how to cook for them?’ (*Participant 209*)

#### Faith

While no question directly asked about participant’s faith or spirituality, participants frequently cited their faith and spirituality in interpreting and coping with one’s cancer diagnosis. This emerged most often as a gratitude to God for their health and well-being at the time of the interview. Trusting in God – on all aspects of the cancer experience – was associated with a positive source of internal motivation and a way to cope.

### Familial level

#### Opinions of and approval by family members

The decision to seek medical care was closely tied to women’s concerns about how poor health would impact their familial roles as wives and mothers. Among married women, the fear of marriage dissolution following a health diagnosis was openly acknowledged as a factor that inhibited individual autonomy to seeking care and treatment.

‘The main barrier in cancer is the husband. Personally, I did not have this problem because I am a widow, I removed a breast and thanks god without needing the authorization and I hope all women don’t listen to their husbands when it comes to their health’. (*Participant 210*)

Concerns about body changes resulting from treatment heightened the need for spousal approval before seeking care, often accompanied by guilt and shame over their reduced ability to fulfil childcare responsibilities. Participants commonly expressed elevated fears regarding their relationships with their spouses. Specifically, concerns about reduced desirability and potential abandonment, specifically following a mastectomy, contributed to delays in care-seeking.

‘She never told her husband until the very last stage because she was afraid, he would marry another woman. We live in a patriarchy. In the Arab world the women take her decision difficultly, disease does not change that’. (*Participant 209*)

‘I know many many women, even some who died, who suffered because their husband was not looking at them the same way’. (*Participant 201*)

### Community level

#### Cancer perspectives and taboo

Such concerns were also reflected in the pervasive socio-cultural affiliations of cancer as a ‘taboo’ illness. Participants frequently evoked how the general attitudes around cancer equated a cancer diagnosis with death.

‘The first trauma the patient is when they receive the diagnostic. It terrorizes the patient emotionally. Just the sound of the word ‘cancer’ is terrorizing’. (*Participant 107*)

These negative societal perspectives and attitudes towards cancer were uniquely heightened among women and associated with gendered aspects of societal value and social capital. For instance, beliefs around cancer aetiology and the associations of cancer treatment with disfigurement (mastectomy and loss of femininity and beauty) manifested in stigma and trauma specific to women’s cancers. As a result, socio-cultural-encouraged modesty among women against discussing personal health burdens, fatalism, and cancer stigma hindered care- and treatment-seeking prior to and following a cancer diagnosis.

‘There are cultural boundaries, we fight against the wrong ideas. Some people think there is a male cancer and a female one and if it’s a female it cannot be cured’. (*Participant 102*)

Participants cited improvements in societal perspectives following the work of civil society, awareness campaigns, and the work of the Lalla Salma Foundation for Cancer Prevention and Treatment. However, there remains a significant need to continue to dismantle misinformation in Morocco around cancer, particularly of cancer fatalism and taboo. Specifically, the conflation of cancer as a deadly disease with no hope of recovery among participants’ family members contributed to the marginalization of cancer patients. Even among participants well-informed of available treatment options and prognoses, such widespread beliefs and norms surfaced in residual personal fears of death and social isolation.

‘The way society looks at a woman affected by cancer. Cancer for us is death, and a woman who has cancer is considered dead’. (*Participant 206*)

#### Acceptance of traditional healers and medicines

Participants noted that acceptance of traditional healers for cancer treatment was common in some communities. Particularly in rural areas with no close health centre, traditional healers were the first sought following symptom presentation or an additional source of medicines and healing to biomedical treatment per the endorsement from other community members, such that, one participant revoked traditional healers and medicines as profiting off the desperation of women cancer patients (*Participant 102*).

### Health system

#### Navigating the healthcare system

Long waiting times and unclear referral pathways were significant barriers to receiving and continuing cancer treatment. Participants described feeling confused and frustrated while navigating the health system, particularly in transitioning from primary care to specialized centres.

‘Women don’t know where they should go, to whom, they can wait 3 months to 6 months just running around without the exact information’. (*Patient 104*)

A key contributing factor was limited health literacy, which hindered their understanding of their diagnosis and treatment plan. Many women viewed their role as passive during medical visits, often refraining from asking clarification questions. This tendency was reinforced by healthcare provider recommendations. Participants recalled being advised to seek care in private rather than public clinics, as providers cited that the innovative medicines and specialized expertise were available exclusively in private facilities. Collectively, prolonged appointment wait times in the public sector, uncertainty about the next steps in care, and external pressures to pursue treatment from private clinics drove patients towards the private healthcare sector.

‘Women face a huge financial burden (very firmly) to get this medication, to get all the additional tests. Even if they manage to get an appointment, they give them so far away so they rather do it in the private sector but it’s so expensive’. (*Participant 202*)

As a result, participants highlighted the financial burden of treatment due to higher out-of-payment costs in the private healthcare sector. To cover these expenses, participants borrowed money from friends and extended family, sold their homes and personal belongings, or chose to forgo certain treatments.

‘It’s not just medication, it’s tests, transportation etc. Even when they can afford it, they spend all their money on cancer. Some sold their house, their gold’. (P*articipant 206*)

#### Health insurance coverage

Many participants recognized the impact of barriers related to care access, availability, and affordability. Financial constraints – including the cost of care, medications, and transportation and stay during treatment – were among the most frequently cited challenges. Participants described how unstable and inadequate medication supply, in part due to weaknesses in government oversight and procurement, posed a significant barrier to treatment adherence. One participant recalled frequently giving away her medications to those less fortunate during shortages (*Participant 207*).

‘First of all, we have a huge problem with medications in the public hospital and even private clinics. We need a monitoring unit in the minister that controls medication stocks’. (*Participant 202*)

Despite compulsory health insurance requirements for all Moroccan citizens, significant inequity in treatment access persists. For members of the poorest households, the government’s social health insurance plan, RAMED, covers key medications, and generalist and specialist consultations in the public healthcare sector. However, participants noted that RAMED coverage was insufficient. The high cost of oncological care often exceeded RAMED coverage and households faced additional non-medical expenses, such as transportation to appointments and housing during treatment and recovery. For women insured by the compulsory health plan, gaps in coverage for specialized tests and treatment led to significant out-of-pockets costs, even among households with greater financial means. Unclear filing protocols for medication reimbursements barred women from fully benefiting from the government’s social protection systems.

#### Support systems

Participants expressed receiving social support through relationships with friends, family members, and other patients, and during positive interactions with healthcare workers. They emphasized that support during the treatment process – especially from other cancer patients or cancer survivors – served as foundational sources of emotional and psychosocial encouragement and inspiration to adhere to treatment.

‘My sisters, they call me to cheer me. I came to the 6th session and I told everyone that I am quitting, I don’t want it anymore, I’d rather die. So, my sisters told me “Don’t think about anything else.”’ (*Participant 201*)

Additionally, participants acknowledged the differences and limitations of familial support compared to that of other patients and friends, attributing these differences to cancer-related knowledge, shared experiences, and solidarity formed through enduring treatments together.

‘Trust me, every patient I met and had a supportive friend they didn’t suffer as much as the other did. The friend is always here. It’s wonderful [shaking voice]. The brother’s love, the husband’s love cannot change because it’s special but it’s nature. Friendship, in opposite, is different’. (*Participant 203*)

#### Patient-provider interactions

While participants respected the expertise of healthcare providers, they also described unsupportive – at times negative – interactions with them. Many participants attributed limitations in provider rapport due to high demand on providers and time constraints they faced. These feelings were endemic; patients readily accepted a limited understanding of their illness and treatment. As a result, participants abided by provider time constraints by reducing the number of questions for health professionals.

‘In this sickness, even if you go to the Doctor, a professor at best, he (/she) won’t be able to give you enough time to explain you the sickness and what is waiting for you’. (*Participant 101*)

Other times, participants recalled strikingly negative experiences with healthcare providers, especially those specializing in cancer. Participants cited the need for greater healthcare providers’ awareness of patient concerns (pain, distress, and fear), increased patience during appointments, and more patient- and person-centred communication, particularly when delivering a cancer diagnosis.

‘Also in doctors training, I think doctors cure diseases but don’t cure the patient, they study 7 years or 14 years with a specialty but few of them actually know how to talk properly to a patient for whom cancer equals death’. (*Participant 102*)

#### Role of LHA and civil society in survivorship

Participants most frequently highlighted the role of cancer civil society and lay health advisors in their personal survivorship journey. In the Moroccan healthcare setting, support network provided by ancillary organizations was often the sole source of survivorship care.

‘Association helps a lot with cancer education. Once you tell a patient “I have been through this,” it relieves them. So many women refused treatment, refused mastectomy but our own experience helped them make the right decision’ (*Participant 209*)

Additionally, participants remarked that their motivation to serve as a LHA or engage in cancer civil society stemmed from their personal cancer experiences and strong determination to give back to other women and cancer patients. Their firsthand experiences were essential in contextualizing the psychosocial implications of treatment amidst patient values and concerns. While their role in cancer advocacy work varied, all participants identified barriers such as a lack of governmental prioritization, low public awareness, gaps in funding, poor coordination between the medical and non-medical cancer sectors to cancer, and limited recognition of their work by healthcare providers.

‘Nationally there was a huge effort done to shift mindsets on cancer. We are progressing but currently we are quite decreasing our involvement. I think the impact is still here of course but I think we shouldn’t stop mobilizing ourselves towards awareness.’ (*Participant 203*)

## Discussion

This qualitative study examined the barriers and facilitators women face in seeking cancer care and during treatment in Morocco. As beneficiaries of care and active stakeholders, cancer survivors involved in the lay health cadres or civil society contribute valuable dual perspectives to national cancer control efforts. Specifically, inputs from participants of the first training programme for cancer lay health advisors in Morocco and women leaders in cancer civil society inform opportunities for greater multisectoral coordination in national cancer control efforts.

Overall, our findings are in line with studies in the Middle East and Northern Africa (MENA) region that examined the major factors related to beliefs, knowledge, and culture that impact uptake of screening and treatment among women (Bowser *et al.*, 2017, p. 201; Sharma *et al.*, 2012; Soliman *et al.*, 2019). Particularly, for cancers of the breast and cervix, early presentation to healthcare services is critical to preventing avoidable suffering and deaths through pathways of downstaging and greater treatment effectiveness (Denny *et al.*, 2017).

At the individual level, barriers to seeking medical care were primarily driven by lack of cancer-specific knowledge, fear of death, and cultural taboo. Similarly, other studies have reported that women often delay care-seeking until physical symptoms become unmanageable (Soliman *et al.*, 2019). This pattern reflects the pervasiveness of negative cancer beliefs and misconceptions, even among women aware of alarm symptoms, barriers that are more frequently reported in LMICs than in high-income countries (Brand *et al.*, 2019). Such delays contribute to a cycle of cancer fatalism and low survival rates, further reinforcing negative beliefs about cancer (Hiom, 2015; Powe and Finnie, 2003). Indeed, prolonged intervals before seeking initial care have been identified as a contributing factor in 70% of global cancer mortality, with an estimated 28.9% of breast cancer cases in Morocco diagnosed at advanced stages (Bray *et al.*, 2018; Khalis *et al.*, 2016). In this study, individual interviews underscore the specific needs for culturally relevant education interventions that mitigate cancer-related stigma while addressing fears and taboos. Participants reported using social media platforms, such as WhatsApp, as information sources, highlighting opportunities to leverage multiple communication channels for educational programming particularly in underserved areas.

Another barrier to cancer care was the influence of family members and community perspectives on when and how to seek medical care. The prevalence of gendered barriers in decision-making to seeking care indicates that cancer control efforts that focused solely on increasing screening and diagnostic services are insufficient. First, a woman’s family and community are significant factors in decision-making to seek care (Alhurishi *et al.*, 2011; Berraho *et al.*, 2012; Soliman *et al.*, 2019). Care-seeking and treatment decisions require women to weigh socio-cultural expectations surrounding their household roles, often manifesting as the need for spousal approval to seek care and finance treatment. Deeply ingrained values regarding women’s bodies as symbols of modesty and femininity coupled with the fear of treatment-related physical disfigurement and spousal rejection or abandonment further shaped these decisions. Second, participants emphasized the need to educate children and younger generations on recognizing cancer alarm symptoms. Mothers, particularly those with less education, could indirectly benefit from school-based cancer education programmes through their children (Uddin *et al.*, 2012). These findings highlight opportunities to scale national awareness and community education programmes within families and communities. While similar gaps have been identified in studies conducted in Morocco, further research is needed to examine the retention of information among school-aged children and its impact on earlier care-seeking behaviours (Soliman *et al.*, 2019).

At the health system level, one of the most frequently described barriers in this study and others were treatment costs and geographical access (Alhurishi *et al.*, 2011; Bowser *et al.*, 2017; Soliman *et al.*, 2019). These barriers arose at multiple points: travelling to the clinic, housing during the period between treatments, and price of medications, tests, and healthcare services. In Morocco, national health insurance schemes partially or fully cover medical costs in the public healthcare system. However, indirect costs incurred outside of the treatment itself (e.g., transportation, stay) placed significant financial burden on patient’s families. Health aimed at reducing financial barriers and preventing impoverishing expenditures should focus on educating patients on how to file for insurance reimbursement, ensuring that national health insurance sufficiently covers medications and care services; coordinating with pharmaceutical companies to increase the drugs’ affordability for both patients and the government; extending services to hard-to-reach populations by supporting low-cost transportation and housing options; and expanding points of contact through nodal cancer units, such as mobile clinics.

Additionally, other reported health system barriers included long waiting times and complex referral pathways, particularly from primary care to specialized cancer treatment centres. Our findings show how confusion in navigating the health systems was compounded by poor patient- and person-centred care, where the disease, and at times, provider incentives for profits in the private sector, took precedence over attending to patient values and concerns. Training healthcare providers in patient- and person-centred communication can improve perceived provider interests and attention to patient needs. In this population, provider counselling can be improved by incorporating cultural values and recognizing that patient illness experiences are influenced by gendered roles and values, faith, and other health belief systems, such as the use of traditional medicines common in Middle Eastern cultures (Ben-Arye *et al.*, 2012). Monitoring systems, such as patient-reported experience measures and patient-reported outcome measures, can enhance health system accountability by integrating routine data collection on health outcomes and patient experiences (Ilbawi *et al.*, 2017).

While concerted efforts under the NCPCP have significantly expanded health resources and trained healthcare providers, gaps remain in providing holistic support to patients navigating cancer diagnosis and treatment pathways. Participants emphasized the support from other cancer patients (e.g., ‘sisters’) and patient advocates as vital sources of strength during treatment. In Morocco, as in other LMICs, limited yet overburdened health resources pose a major challenge in service delivery (Khalis *et al.*, 2021). Adopting a collaborative approach to addressing these limitations can improve patient experience and satisfaction – hallmarks of high-quality care (Kruk *et al.*, 2018). Our results highlight the benefit of strengthening the alternative oncology workforce and fostering collaborations across civil society sectors, which presents promising opportunities to enhance comprehensive cancer control efforts (World Health Organization, 2013). In this study, the Patient-Partner programme exemplifies an alternative auxiliary cadre to address gaps in psychosocial and integral care. While LHAs are an evidence-based model for cancer care delivery, greater support is needed to facilitate their implementation in LMICs (Rodriguez *et al.*, 2017).

The findings from this study are within the context of several limitations. First, our study used a targeted sampling to recruit participants with similar lived experiences (previous or current cancer patients and active members of civil society). Therefore, the thematic patterns may be different for other populations and contexts. Second, qualitative data were collected a few months after the conclusion of the Patient-Partner programme. For some, this was their first and primary source of exposure to civil society work. Therefore, we recommend further research following participant outcomes one year or more after completing the Patient-Partner training and certification programme.

Despite these limitations, the study offers several important strengths. First, to the best of our knowledge, this is the first study to explore the rich, intersectional experiences of female Moroccan cancer survivors and LHAs or civil society members. The perspectives of these participants provide a unique dual insight into the barriers and facilitators faced by former cancer patients, enriched by their in-depth understanding as active LHA or civil society members. Second, our findings capture multiple dimensions of the alternative cancer workforce, sampling participants from Morocco’s first lay health advisor training and certification programme as well as members from multiple civil society cancer organizations. Third, participants included survivors of various cancer types, expanding upon the only other qualitative study in Morocco, which examined the perspectives of breast cancer patients.

### Conclusions & recommendations

Our findings underscore the importance of addressing the multilevel factors in cancer care and control from the perspectives of LHAs and civil society member – who are themselves cancer survivors. This includes addressing the identified barriers and facilitators to women’s cancer care and treatment at the individual patient, family, community, and health system levels, highlighting multiple pathways to improve women’s cancer and control in Morocco.

Based on our findings, investment and interventions aimed at improving women’s cancer care and control must reconcile the barriers within and beyond the health system. Within the health system, efforts should focus on mitigating the indirect costs of cancer care (e.g., travelling and stay), simplifying navigating the healthcare system (e.g., public and private), and improving access to insurance and social assistance programmes. At the community level, culturally relevant cancer education can help counter longstanding stigmatizing cancer characterizations, including gendered misinformation, taboo, and shame surrounding women’s cancers. A holistic approach to supporting patients – such as through the LHA cadre and civil society efforts – presents an opportunity for interdisciplinary collaborations to improve overall patient well-being. Future research is needed to evaluate the impact of the LHAs on improving patient experiences and satisfaction.

## Supporting information

Luo et al. supplementary material Luo et al. supplementary material

## Supplementary material

The supplementary material for this article can be found at https://doi.org/10.1017/S1463423625100169
