# Supplementary material for: Challenges to women’s cancer control in Morocco: a qualitative study of lay advisors and civil society perspectives
Source: Prim Health Care Res Dev. 2025 Jun 16;26:e47. doi: 10.1017/S1463423625100169 (PMC12175096; doi:10.1017/S1463423625100169)
Supplement: Luo et al. supplementary material [file S1463423625100169sup001.docx]

​**Appendix 1. Interview Topic Guide**

**Part 1. Protecting Interviewee Privacy and Confidentiality**

1. Are you okay with recording this interview?

**Part 2. Topic Guide and Probing Queestions**

**Questions 1-11:** Evaluation of the Patient-Partner Program & Association work

*Experience in the Patient-Partner Program*

1. How did you hear about the Patient-Partner in Cancer Care program?
2. What motivated you to participate in the Patient-Partner program?
3. Can you give a summary of the Patient-Partner program?
4. What are important and key areas that you have learned from the program? Please be specific about lessons, workshops, skills that you feel are important?

*Using Experiences as a Patient-Partner to Strengthen Cancer Association Work*

1. How long have you been invovled in cancer association work?
2. Please describe the work of the cancer association? What do you work on?
3. Which parts of the program have you found to be most beneficial for your cancer association work?
4. How have you been able to use your training as a Patient-Partner in your cancer association work?
5. What motivates you to be involved in your cancer association work?
   1. What do you work on in your cancer association work?
   2. How does this work align with your personal goals?
6. Please describe challenges you face in your cancer association work?
7. How has *all* cancer association work (not just your own work) improved cancer?

**Questions 12-19:** Facilitators and Barriers to Cancer Care among Women patients in Morocco

*How Cancer Care, Education, and Prevention can be Improved*

1. From your own experience and interacting with other female patients, what are obstacles that impact a women’s access to health care?
   1. Can you describe any barriers for women?
   2. Are women able to make the decision by themselves? If no, do women consult?
   3. What concerns or fears a women may have?
2. What are other sources do women get information about their health?
   1. From members, friends, other women?
3. How do women get information about cancer?
   1. How can cancer education be improved?
4. How can support for women during cancer treatment and recovery be improved?
   1. By family members
   2. By healthcare providers
   3. By others (please specify)
5. What areas in cancer care and education need to be improved? In what ways and why?
6. Overall, how do you feel that cancer association work can be improved?
7. Please describe how the Patient-Partner program can be improved to best benefit participants in their cancer association work?
8. Is there anything else you would like to add?

**Questions 20-27:** Sociodemographic Characteristics

1. How old are you?
2. What type of cancer did you previously have? How long was your treatment?
3. Are you currently married? If no, are you currently living with a man, widowed, divorced, separated, never married, or other? If other, please specify.
4. How many children do you have?
5. How many people live in the same house as you?
6. What is your highest level of education that you have completed – secondary, college, higher, or other? If other, please specify.
7. What is your current occupation?
8. Do you earn less than 3,000 dirham, between 3,000-8,000, or greater than 8,000 dirham per month?
